# Supplementary material for: Cholesterol Metabolic Profiling of HDL in Women with Late-Onset Preeclampsia
Source: Int J Mol Sci. 2023 Jul 12;24(14):11357. doi: 10.3390/ijms241411357 (PMC10380085; doi:10.3390/ijms241411357)
Supplement: Supplementary file 1 [file ijms-24-11357-s001.zip › ijms-2417803-supplementary.pdf]

Table S1. Changes in lipid profile parameters in RG and PG in the 1st and 2nd trimester

	1st trimester		2nd trimester		p_1	p_2
	RG (N=70)	PG (N=20)	RG (N=70)	PG (N=20)		
WG	12.8 ± 0.80	12.7 ± 0.16	23.3 ± 0.83	23.4 ± 0.99		
Age, years	32.0 ± 5.56	33.1 ± 4.26				
MAP, mmHg	85.9 ± 11.59	92.4 ± 9.01[dg]	82.5 ± 11.51	89.8 ± 9.64[dg]	**<0.05**	0.164
BMI[b], kg/m^2	23.6 (21.1-27.5)	27.5 (24.2-31.1)	25.4 (23.6–30.0)	28.4[eg] (26.4–32.7)	**<0.001**	**<0.001**
Weight gain[b], kg	2.15 (1.27-4.07)	3.00 (0.25-4.40)	5.40 (3.72–7.02)	4.70 (2.82–6.75)	**<0.001**	**<0.05**
Weight gain[b], %	4.00 (2.00-6.00)	4.00 (2.25-5.90)	7.00 (5.00–9.75)	5.50 (4.00–8.75)	**<0.001**	**<0.05**
TC, mmol/L	5.33 ± 1.070	5.46 ± 0.762[dg]	6.81 ± 1.365	6.61 ± 1.236	**<0.001**	**<0.001**
HDL-C, mmol/L	1.77 ± 0.336	1.90 ± 0.587	2.11 ± 0.386	1.88 ± 0.363[dg]	**<0.001**	0.885
LDL-C, mmol/L	2.94 ± 0.849	2.81 ± 0.633	3.81 ± 1.148	3.61 ± 1.104	**<0.001**	**<0.001**
TG[a], mmol/L	1.27 (1.18-1.38)	1.53 (1.27-1.85)	1.86 (1.73–2.00)	2.33[dg] (2.00–2.71)	**<0.001**	**<0.001**

RG – risk group; PG – preeclampsia group; WG – week of gestation; BMI – body mass index; MAP – mean arterial pressure; TC – total cholesterol; HDL-C – high density lipoprotein cholesterol; LDL-C – low density lipoprotein cholesterol; TG – triglycerides

Data are shown as mean ± standard deviation; [a] geometric mean (95th CI); [b] median (interquartile range)

p_1 – Paired-Samples T-Test for risk group; p_2 –for preeclampsia group

p_1^c – Wilcoxon Test for risk group; p_2^c –for preeclampsia group

Difference significantly different from the risk group: [d] Analysis of covariance (ANCOVA); [e] Non-parametric ANCOVA (Quade method)

[f] p<0.001; [g] p<0.05
